# Editorial: Network science approaches to risk assessment of mental disorders and dementia

**DOI:** 10.3389/fpsyt.2023.1286227

**Published:** 2023-11-28

**Authors:** Zhongzhi Xu, Wentian Li, Xiaoying Tang, Mingyang Li, Paul S. F. Yip, Qingpeng Zhang

**Affiliations:** ^1^School of Public Health, Sun Yat-sen University, Guangzhou, China; ^2^Wuhan Hospital for Psychotherapy, Tongji Medical College of Huazhong University of Science and Technology, Wuhan, China; ^3^Department of Electronic and Electrical Engineering, Southern University of Science and Technology, Shenzhen, China; ^4^Department of Industrial and Management Systems Engineering, The University of South Florida, Tampa, FL, United States; ^5^Hong Kong Jockey Club Centre for Suicide Research and Prevention, Faculty of Social Sciences, The University of Hong Kong, Hong Kong, Hong Kong SAR, China; ^6^Department of Pharmacology and Pharmacy, The University of Hong Kong, Hong Kong, Hong Kong SAR, China; ^7^Musketeers Foundation Institute of Data Science, The University of Hong Kong, Hong Kong, Hong Kong SAR, China

**Keywords:** network science, mental disorder, dementia, machine learning, risk assessment, artificial intelligence

As our understanding of mental disorders advances, it becomes increasingly clear that many conditions are not simple linear cause-effect relationships (1, 2). The burgeoning field of network science offers powerful tools for understanding and addressing such issues (3). Moving away from traditional methods, network analysis allows researchers to focus on the complex relationships between individual variables, thus providing a nuanced understanding of the intricate structures behind mental health conditions (4, 5). In this Research Topic, we present four articles that employ network analysis to explore critical questions related to mental health.

In the first article, “*Understanding MMPI-2 response structure between schizophrenia and healthy individuals*,” Hsu et al. seek to disentangle the cognitive and affective aspects of depression. The findings suggest that core symptoms such as “hopelessness” and “anxiety” could serve as critical intervention points, echoing the broader discourse on individualized treatment in mental health care.

The second article, “*Abnormal temporal variability of rich-club organization in three major psychiatric conditions*,” authored by Niu et al., delves into the association between social media use and mental health. Through network analysis, the study unveils the centrality of “social comparison” and “negative feedback,” indicating the importance of these factors in initiating or perpetuating anxiety and depression.

Wang et al. leads the third article, titled “*The association between family relationships and depressive symptoms among pregnant women: a network analysis*.” This paper offers invaluable insights into the intersection of family relationships and depressive symptoms in pregnant women. Notably, the study identifies “equal status with husband” and “couple relationship” as central nodes in the network, highlighting the necessity of targeted interventions for these relationships to alleviate depressive symptoms.

The fourth article, by Jing et al., is titled “*Social, lifestyle, and health status characteristics as a proxy for occupational burnout identification: a network approach analysis*.” This study unravels the interconnected risk factors associated with occupational burnout. Key demographic and lifestyle variables are identified, such as age and dietary habits, which can serve as proxies for assessing the likelihood of burnout in workplace settings.

Collectively, these articles showcase the versatility and applicability of network analysis in unraveling the complexities of mental health issues. They extend our understanding of how individual symptoms, social factors, and lifestyle choices interact to contribute to mental health conditions. These works suggest that addressing central nodes in a network could be a more effective strategy for prevention and intervention, a perspective that offers new pathways for future research and practical applications.

By bringing these disparate but interconnected topics under the aegis of network analysis, we aim to inspire more multidisciplinary efforts that leverage this potent analytical tool. Through its granularity and precision, network analysis can empower researchers and practitioners alike to develop more targeted, impactful, and humane approaches to mental health care.

## Author contributions

ZX: Writing – original draft, Writing – review & editing. WL: Writing – review & editing. XT: Writing – review & editing. ML: Writing – review & editing. PY: Writing – review & editing. QZ: Writing – review & editing.
